# Neuroinflammation and Brain Functional Disconnection in Alzheimer’s Disease

**DOI:** 10.3389/fnagi.2013.00081

**Published:** 2013-11-25

**Authors:** Francesca Baglio, Marina Saresella, Maria Giulia Preti, Monia Cabinio, Ludovica Griffanti, Ivana Marventano, Federica Piancone, Elena Calabrese, Raffaello Nemni, Mario Clerici

**Affiliations:** ^1^IRCCS, Don Gnocchi Foundation, Milan, Italy; ^2^Department of Electronics, Information and Bioengineering, Politecnico di Milano, Milan, Italy; ^3^Università degli Studi di Milano, Milan, Italy

**Keywords:** Alzheimer’s disease, mild cognitive impairment, magnetic resonance imaging, diffusion tensor imaging, immunology, neuroinflammation

## Abstract

Neuroinflammation and brain functional disconnection result from β-amyloid (Aβ) accumulation and play fundamental roles in the pathogenesis of Alzheimer’s disease (AD). We investigated possible correlations between these two AD-associated phenomena using DTI-based tractography and immunologic analyses in people with amnestic mild cognitive impairment (aMCI) and AD. DTI-Analyses focused on corpus callosum (CC). We found that frontal CC regions were preserved with respect to the posterior ones in aMCI; in these individuals significant correlations were seen between DTI-derived metrics in frontal-parietal CC areas and Aβ_42_-stimulated BDNF-producing CD4+ T lymphocytes and PDL-1-expressing CD14+ cells. These associations were lost in AD where DTI data involving the same CC areas correlated instead with Aβ_42_-stimulated interleukin (IL)-21 producing CD4+ T lymphocytes. Higher susceptibility to PDL-1-mediated apoptosis of Aβ_42_-specific lymphocytes and BDNF-associated survival of existing neurons could contribute to the relative CC structure preservation seen in aMCI. These potentially protective mechanisms are lost in frank AD, when severe alterations in the CC are mirrored in peripheral blood by proinflammatory cytokines-producing T cells. Monitoring of immune cells in peripheral blood could have a prognostic value in AD.

## Introduction

Alzheimer’s disease (AD) is a neurodegenerative disorder involving both gray matter (GM) and white matter (WM) tissues that is now well defined as a part of a *continuum* of clinical and biological phenomena. AD is characterized by the accumulation of amyloid-beta (Aβ) peptide into amyloid plaques in the extracellular brain parenchyma and by the formation of neurofibrillary tangles (NFT) within neurons as a result of the abnormal phosphorylation of the microtubules-associated tau-protein. Neuroinflammation is strongly suspected to play an important role in AD that might be the culprit of the disease or, possibly, a reaction to the pathology (Ke et al., 2006; Zipp and Aktas, 2006). Thus, both increased concentrations of proinflammatory cytokines (Bauer et al., 1991; Strauss et al., 1992; Remarque et al., 2001; Bermejo et al., 2008) and changes in lymphocyte subsets, with an augmented percentage of activated immune cells are described in AD (Lombardi et al., 1999; Speciale et al., 2007; Saresella et al., 2011). In particular, recent results indicated that a significant reduction of PD-1- and PDL-1-expressing cells is present in AD and amnestic mild cognitive impairment (aMCI), a prodromal stage of AD. The interaction between these molecules is responsible for the induction of tolerance and for the apoptosis of antigen-specific cells (Francisco et al., 2010); as the impairment seen in AD and aMCI is specific for Aβ-stimulated cells, this alteration could play a role in the neuroinflammation accompanying AD (Saresella et al., 2012).

Functional disconnection as a consequence of Aβ pathology has also recently been hypothesized to be a major mechanism in AD clinical evolution (Drzezga et al., 2011). This suggestion stems from the evidence that WM damage can be observed even in AD patients in early stages of disease, and this correlates with clinical measures of cognitive disability (Parente et al., 2008; Bozzali et al., 2011). DTI-based tractography is an ideal MRI based technique for the study of WM changes in the most relevant fiber bundles of the brain (Le Bihan, 2003), and DTI results of previous studies confirmed that brain disconnection plays an important role in AD pathophysiology, and contributes to the progressive accumulation of disabilities in the transitional stage between normal aging and dementia (Bozzali et al., 2002, 2011; Takahashi et al., 2002; Duan et al., 2006; Naggara et al., 2006; Zhang et al., 2007; Serra et al., 2010; Gili et al., 2011).

The corpus callosum (CC), the largest WM fiber connection of the human brain, is an ideal target to test physiopathological changes in AD. In a previous DTI-study (Preti et al., 2012), we investigated the pattern of CC abnormalities in different stages of AD and showed the presence of an early selective damage of the central and posterior CC subregions in this disease. This CC damage pattern is consistent both with the distribution of GM loss found in volumetric studies (Karas et al., 2008; Kakeda and Korogi, 2010; Vemuri et al., 2011), and with the neuropsychological profile of the patients (Leube et al., 2008; Risacher et al., 2009; Serra et al., 2010). These alteration are also consistent with the pathological knowledge that we have of AD progression, according to which the posterior CC subregions are preferentially involved in the earlier stages of AD whereas and the anterior CC subregions are usually affected in the later stages of disease (Brun and Englund, 2002).

We investigated possible correlations between neuroinflammatory parameters and brain disconnection in AD and aMCI using DTI-based tractography and immunologic analyses performed on peripheral blood mononuclear cells. In particular, the CC was evaluated in its different parts to identify the anatomical regions in which the damage mediated by inflammation plays a prominent role, and immunologic studies were performed in peripheral blood in the attempt to identify easily reproducible diagnostic and/or prognostic parameters.

## Materials and Methods

### Subjects

Forty patients diagnosed with probable AD according to the NINCDS-ADRDA criteria (McKhann et al., 2011) and 20 subjects diagnosed with aMCI according to Petersen criteria (Petersen, 2004) were included in the study. All subjects were consecutively recruited at the Fondazione Don Gnocchi, IRCCS in Milano, Italy. AD patients were in mild stage of the disease as determined by both clinical dementia rating (CDR) (Morris, 1993) scale (CDR range 0.5–1.5) and mini-mental state examination score (Magni et al., 1996) (MMSE mean/SD 20.2/2.7). To be eligible for the study, aMCI individuals were required to meet the Grundman operational criteria (Grundman et al., 2004): memory complaint, confirmed by an informant; abnormal memory function, documented by previous extensive neuropsychological evaluation; normal general cognitive function, as determined by both CDR scale (Morris, 1993) (CDR with at least a 0.5 in the memory domain) and MMSE (Magni et al., 1996) score (MMSE greater than or equal to 24); no impairment in functional activities of daily living as determined by a clinical interview with the patient and informant; no significant cerebral vascular disease (Hachinski score less than or equal to 4) (Rosen et al., 1980); no major psychiatric illnesses with particular attention to exclude subjects with history of depression (Hamilton Depression Rating Scale score less than or equal to 12) (Hamilton, 1960). To increase the diagnostic accuracy analyses of hippocampal volumes, an index of downstream neural injury according to the guidelines for MCI due to Alzheimer’s dementia (Sperling et al., 2011), were also included in the study. Demographic details of the study sample are summarized in Table 1. Twenty-five healthy controls (CTRs) that were age-and sex-matched to the AD patients (70.2 ± 5.1 years; 11M/14F; MMSE score >26) were also included in the MR analysis for the tractographic atlas construction. The study conformed to the ethical principles of the Helsinki Declaration; all patients or their care-givers gave informed consent according to a protocol approved by the local ethics committee of the Don Gnocchi Foundation.

**Table 1:** **Demographical and anatomical information of the sample**.

	AD	aMCI	CTR
N	40	20	25
Age [years, mean (SD)]	73.7 (12)	73.9 (5.4)	70.2 (5.1)
Sex (M:F)	17:23	12:8	11:14.
MMSE score [mean (SD)]	20.2 (2.7) (§)	25.6 (1.7) (^∧^)	29.1 (0.7) (#)
CDR (range)	1–1.5 (§)	0–0.5	0
Right hippocampal volume [mm^3^, mean (SD)]	2898 (575) (§)	3158 (519) (^∧^)	3774 (558) (#)
Left hippocampal volume [mm^3^, mean (SD)]	2719 (482) (§)	3065 (489) (^∧^)	3607 (450) (#)

AD, Alzheimer’s disease; aMCI, amnestic mild cognitive impairment; CTR, healthy controls; MMSE, mini-mental state evaluation; CDR, clinical dementia rating scale; SD, standard deviation. Chi-squared test was used for gender comparison and one-way ANOVA with Bonferroni *post hoc* test was used for age, MMSE score and hippocampal volumes comparisons (significance level: *p* < 0.05). (#) Significant compared to MCI and AD group; (§) significant compared to MCI and CTR; (∧) significant compared to AD and CTR.

### MRI acquisitions and analyses

#### MRI acquisition protocol

Brain MR images were acquired using a 1.5-T scanner (Siemens Magnetom Avanto, Erlangen, Germany). The following sequences were acquired: (1) dual-echo turbo spin echo (TR/TE = 2920/22 ms, FoV = 240 mm × 180 mm, in-plane resolution = 0.75 mm × 0.75 mm, slice thickness = 4 mm, number of axial slices = 25) and FLAIR sequence (TR/TE = 9000/121 ms, FoV = 240 × 168 mm, in-plane resolution = 0.94 mm × 0.94 mm, slice thickness = 5 mm, number of coronal slices = 24), to exclude patients showing WM hyperintensities outside the normal range; (2) three-dimensional T1-weighted magnetization prepared rapid gradient echo (MPRAGE) (TR/TE = 1900/3.37 ms, FoV = 192 mm × 256 mm, in-plane resolution 1 mm × 1 mm, slice thickness = 1 mm, number of axial slices = 176); (3) diffusion-weighted pulsed-gradient spin-echo planar (TR/TE = 7000/94 ms, FOV = 320 mm × 240 mm, in-plane resolution = 2.5 mm × 2.5 mm, slice thickness = 2.5 mm, number of axial slices = 50), with diffusion gradients (*b*-value = 900s/mm^2^) applied in 12 non-collinear directions. Two runs of DW images with one *b* = 0 image without diffusion weighting were acquired for each subject.

#### Analysis of T1-weighted structural images

Two different analyses were performed on the T1-weighted structural images in order to provide further information about the underline pathology in this cohort of subjects: (1) computation of hippocampal volumes; (2) voxel-based morphometry (VBM) analysis.

Hippocampal volume data were extracted for each subject from high-resolution T1 3D images. Segmentation of right and left hippocampi was performed using FSL’s FIRST method (Patenaude et al., 2011), an approach combining both shape and intensity information within a Bayesian Model to segment subcortical structures. After hippocampal segmentation, volumetric data had been obtained in each subject using a specific FSL function.

The VBM analysis was conducted using VBM8^1^, toolbox of SPM8^2^, running on Matlab 7.6.0^3^. VBM was conducted according to the Unified Method (Ashburner and Friston, 2005). After GM segmentation all images underwent spatial smoothing using a Gaussian kernel (FWHM 8 mm).

#### Analysis of diffusion-weighted images

Diffusion-Weighted Images (DWI) were first corrected for eddy current distortions, using FSL^4^. For every subject, the images of the second run were registered to the first one using SPM5 (see text footnote 2). The affine transformation between the *b* = 0 image of the second run and the *b* = 0 image of the first one was estimated and applied to all the DWI of the second run. From the DWI, the diffusion tensor (DT) and its scalar invariants fractional anisotropy (FA) and mean diffusivity (MD) were computed for every voxel, using Diffusion Toolkit^5^ v0.6, which firstly rotates the B-matrix for slice angulation and for the rotation applied by FSL and SPM.

#### CC tractographic analysis

To assess CC integrity, the atlas-based tractography method described in Preti et al. (2012) was adopted. For the atlas construction, individual deterministic tractography of the CC was performed for the CTRs with Diffusion Toolkit, using all voxels as seed points (brute force approach) and the Interpolated Streamline algorithm. FA and angle thresholds of 0.2 and 35° respectively were adopted as stopping criteria for the tractographic algorithm. The tracts of seven CC regions portions (CC1: orbital frontal, CC2: anterior frontal, CC3: superior frontal, CC4: superior parietal, CC5: posterior parietal, CC6: temporal, CC7: occipital) were selected from the whole brain tracts by drawing regions of interest (ROIs) on the FA map using Trackvis v0.5.1 (see text footnote 6) and following the ROI positioning suggested by Lebel et al. (2010). From the individual tractographic reconstructions, the probabilistic atlases of the seven CC portions were obtained, adopting the method described in detail in Preti et al. (2012).

Average FA and MD values along the tracts in the seven CC portions (CC1–CC7) were extracted for every subject basing on the locations given by the created tractographic atlases. First, all subjects were linearly registered to the atlas space with FSL, using the FMRIB58_FA as template for the alignment. Then, the estimated transformation between individual FA maps and the template was applied to both FA and MD maps of every subject. Finally, the registered FA and MD maps of all subjects were masked with the atlases and, for each tract portion, the index mean values weighted for the probability given by the atlas were computed.

### Immunological analyses

#### Blood sample collection and cell culture

Whole blood was collected in vacutainer tubes containing ethylenediaminetetraacetic acid (EDTA) (Becton Dickinson & Co., Rutherford, NJ, USA). PBMC were separated on lympholyte separation medium (Cedarlane, Hornby, ON, Canada); viable leukocytes were determined using a Scepter 2.0 Handheld Automated Cell Counter (Millipore, Billerica, MA, USA).

About 1 × 106 PBMC were cultured for 24 h in RPMI 1640 + 10% human serum, 2 mM l-glutamine, and 1% penicillin (Invitrogen Ltd, Paisley, UK) with either non-immunogenic peptides (Saresella et al., 2008) or with 10 μg/ml of Aβ_42_-peptide soluble monomer (Sigma, St Louis, MO, USA) at 37°C in a humidified 5% CO2 atmosphere for 24 h. The dose of peptides was chosen based on previously results (Saresella et al., 2010). For cytokine analyses, 10 μg/ml of brefeldin A (Sigma-Aldrich) was added to the cell cultures during the last 6 h of stimulation to block protein secretion.

#### Flow-cytometry immunofluorescent staining

Aβ_42_-peptide – or non-immunogenic peptides-stimulated PBMC were resuspended in PBS and stained with anti-CD4, -CD8, -CD19, -CD14, -PD-1, or -PDL-1 specific mAbs (eBioscience, San Diego, CA, USA). Cells were washed after 30 min incubation at room temperature in the dark, treated with FIX and PERM (FIX and PERM Cell Permeabilization kits; eBioscience) and incubated for 30 min at 4°C in the dark with cytokine-specific mAbs.

The following mAbs were used: phycoerythrin (PE)-Cyanin-7 (PC7)-labeled-anti-CD4 (clone SFCI12T4D11; mouseIgG1), PE-Cyanin-5 (PC5)-labeled-anti-CD8 (clone SFC12Thy2D3; mouse-IgG_1_), PC5-labeled-anti-CD19 (clone J4.119; mouse IgG_1_), and PC7-labeled-anti CD14 (clone RMO52, mouse IgG_2a_, Beckman-Coulter Brea, CA, USA); PE-labeled-anti-PD-1 (clone MIH4; mouse-IgG_1_) and (PC5)-labeled-anti-PDL-1 (clone MIH1; mouse-IgG1; eBioscience Cornerstone Court, West, San Diego, CA, USA). The intracellular molecule detection mAbs used were: PE-labeled-anti-interleukin (IL)-6 (clone 1936, mouse IgG_2B_), PE-labeled-anti-IL-10 (clone JES9D7; mouse IgG_1_), PE-labeled-anti-IL-22 (clone 142928, mouse IgG1), Fluorescein isothiocyanate (FITC)-labeled-anti-IL-12 (clone 27537, mouse IgG_1_), PE-labeled-anti-TGF-β- (clone 9016, mouse IgG1), PE-labeled-anti-IFNγ (clone 25723, mouse IgG_2b_), and PE-labeled-anti-BDNF (clone 35909; mouse-IgG1 R&D Systems, Inc., Minneapolis, MN, USA); PE-labeled-anti-IL-9 (clone MH9A4, mouse IgG2B,k), PE- labeled-anti-IL-23 (clone C11.5, mouse IgG_1k_), and PC5-labeled-anti-IL-17 (clone BL168, mouse IgG1k; Biolegend San Diego, CA, USA); PE-labeled-anti-ROR-C/γ (clone AFKJS-9, rat IgG2a), PE-labeled- anti T-bet- (clone 39D, mouse IgG1), PE-labeled-anti-GATA-3 (cloneTWAY, rat IgG2B,k), and PE-labeled-anti-IL-21 (clone 3A3-N2, mouse IgG1; Bioscience).

#### Flow-cytometry analysis

A Beckman-Coulter Cytomics FC-500 flow cytometer equipped with a single 15 mW argon ion laser operating at 488 nm and interfaced with CXP Software 2.1 was used. Two-hundred-thousand events were acquired and gated on lymphocyte or monocyte FSC and SSC properties. Samples were first run using isotype control or single fluorochrome-stained preparations for color compensation. Rainbow Calibration Particles (Spherotec, Inc. Lake Forest, IL, USA) were used to standardize flow-cytometry results.

### Statistical analysis

The statistical analyses were accomplished using commercial software (SPSS for Windows, V 18.0; SPSS Inc). We compared aMCI, AD, and CTRs on demographic data, using the chi-square test and one-way ANOVA with Bonferroni *post hoc* test for categorical and dimensional variables, respectively. To test the comparisons between the aMCI and AD groups, Mann–Whitney *U*- or *t*-test were performed and continuous variables were described using median and interquartile range, according to variable skewness. The statistical correlations between immunological parameters and RM data were investigated by means of Spearman correlation coefficient and 95% confidence limits performed by Fisher’s *Z* transformation.

## Results

### Demographical and anatomical characteristics of the participants

CTRs, aMCI subjects, and AD patients were comparable for age and gender; a significant difference was found instead for MMSE and CDR values, accordingly with the adopted inclusion criteria (Table 1).

**Figure 1:**
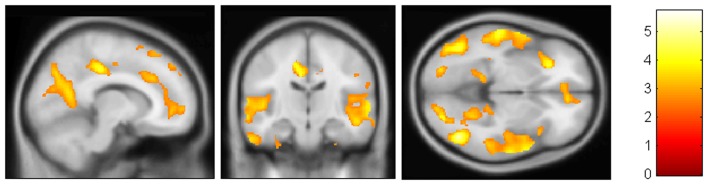
**VBM results**. AD patients were found to be significantly more atrophic than aMCI in medial, anterior, and postero-inferior regions of temporal lobes (left > right) and precuneus/posterior cingulate.(FDR < 0.05)

Structural MRI confirmed the pattern of GM atrophy typical of aMCI and AD. VBM showed that patients with AD were significantly more atrophic than aMCI in several brain regions including medial, anterior, and postero-inferior regions of temporal lobes (left > right) and precuneus/posterior cingulate (Figure 1). Hippocampal volumes of CTRs were significantly different compared to aMCI and AD (*p* < 0.05 Bonferroni *post hoc*; CTRs > aMCI > AD).

### DTI analyses

**Figure 2:**
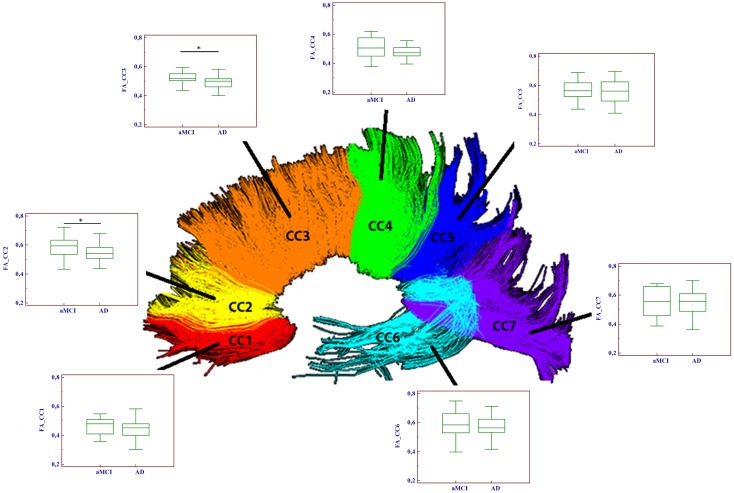
**Comparison of fractional anisotropy (FA) values between aMCI and AD patients in the seven portions of the corpus callosum (CC)**. Significant differences between the two groups were found only in two frontal CC portions (CC2 and CC3). (*p < 0.05).

Significantly reduced FA values were found in the frontal CC regions (CC2–CC3) of AD compared to aMCI subjects (*p* < 0.01). Moreover, MD values were significantly higher in AD with respect to aMCI group, in every portion with the exception of CC7. Results of the comparison between mean FA/MD computed in the seven CC portions of the two groups (aMCI and AD patients) are showed in Table 2 and Figure 2.

**Table 2:** **Results of DTI-based tractography**.

	aAMCI		AD		Comparison aMCI-AD (*)	
CC portion	FA	MD	FA	MD	FA	MD
	Mean (SD)	Mean (SD)	Mean (SD)	Mean (SD)	*p* value	*p* value
CC1	0.47 (0.06)	0.86 (0.11)	0.45 (0.06)	0.94 (0.10)	n.s.	0.013
CC2	0.59 (0.06)	0.90 (0.09)	0.54 (0.07)	0.98 (0.11)	0.009	0.012
CC3	0.52 (0.06)	0.97 (0.07)	0.49 (0.05)	1.04 (0.09)	0.021	0.018
CC4	0.50 (0.07)	1.04 (0.11)	0.48 (0.07)	1.12 (0.13)	n.s.	0.034
CC5	0.58 (0.08)	0.93 (0.09)	0.54 (0.08)	1.03 (0.15)	n.s.	0.009
CC6	0.59 (0.09)	1.01 (0.20)	0.57 (0.09)	1.13 (0.24)	n.s.	0.039
CC7	0.54 (0.10)	0.97 (0.15)	0.53 (0.10)	1.03 (0.13)	n.s.	n.s.

Comparison between DTI-metrics (mean FA/MD) computed in the seven CC portions of the two groups (aMCI and AD patients).AD, Alzheimer’s disease; aMCI, amnestic mild cognitive impairment; CC, corpus callosum; FA, fractional anisotropy; MD, mean diffusivity. (*) *p*-values refer to *t*-test, significance level *p* < 0.05

### Immunological results

#### Cytokine-producing and transcription factor-expressing T cells in Aβ_42_-peptide-stimulated cell cultures

PBMC obtained from AD and aMCI patients were stimulated *in vitro* with either Aβ_42_-peptide or with non-immunogenic control peptides. Cytofluorimetric analyses were used to analyze IFN**γ**-, IL-9-, IL-17-, IL-21-, IL-22-, and BDNF-producing CD4+ and CD8+ T lymphocytes. Results showed that, whereas no differences were seen when cells were stimulated with non-immunogenic peptides (data not shown), Aβ_42_-stimulated-BDNF-producing CD4+ T lymphocytes were significantly reduced in AD compared to aMCI (*p* = 0.003); interestingly, and confirming the increased inflammatory status of AD, IFN**γ**-producing-CD8+ T lymphocytes were increased in AD compared to AMCI individuals (*p* = 0.012).

Distinct transcription factors (TF) are activated during the differentiation of T cells into functional subsets. We analyzed T-Bet, ROR-C/**γ** and GATA-3 TF in all the individuals enrolled in the study. Results show an increase of Aβ_42_-stimulated-CD8+ T cells expressing T-bet in AD compared to aMCI (*p* = <0.001). As T-bet is the TF expressed by cells that have differentiated into TH1 lymphocytes, and because such lymphocytes are characterized by the production of IFN**γ**, these results explain the increased production of this cytokine seen in AD (Table 3).

**Table 3:** **CD4+, CD8+, and CD14+ cells that produce cytokines and CD4+, CD8+, CD14+, and CD19+ cells that express PD-1 or PD-L1 in patients with a diagnosis of either AD or aMCI**.

	aMCI	AD	*p*-value (*)
CD4^+^GATA-3^+^	0.40 (0.00 −1.25)	0.05 (0.00 −0.70)	n.s.
CD4^+^ROR-C-γ^+^	0.16 (0.07 −0.40)	0.10 (0.00 −0.38)	n.s.
CD4^+^IFNγ^+^	0.10 (0.01 −0.32)	0.15 (0.00 −0.40)	n.s.
CD4^+^IL-17^+^	0.05 (0.00 −0.08)	0.07 (0.00 −0.29)	n.s.
CD4^+^IL-9^+^	0.12 (0.04 −0.40)	0.10 (0.02 −0.22)	n.s.
CD4^+^IL-21^+^	0.01 (0.00 −0.73)	0.30 (0.02 −0.62)	n.s.
CD4^+^IL-22^+^	0.20 (0.10 −0.50)	0.42 (0.10 −0.70)	n.s.
CD4^+^BDNF^+^	0.39 (0.27 −0.58)	0.12 (0.03 −0.29)	0.003
CD8^+^BDNF^+^	0.45 (0.18 −1.71)	0.31 (0.13 −0.55)	n.s.
CD8^+^T-bet^+^	0.01 (0.00 −0.05)	1.22 (0.09 −3.16)	<0.001
CD8^+^IFNγ^+^	0.02 (0.00 −0.065)	0.065 (0.01 −0.15)	0.0127
CD14^+^TGF-β^+^	5.50 (2.27 −16.05)	1.87 (0.53 −3.05)	0.003
CD14^+^IL-6^+^	15.50 (1.27 −38.77)	10.11 (2.20 −16.00)	n.s.
CD14^+^IL-10^+^	3.95 (2.43 −13.88)	2.05 (0.53 −12.82)	n.s.
CD14^+^IL-12p35^+^	0.6 (0.00 −1.82)	0.18 (0.02 −1.4)	n.s.
CD14^+^IL-23^+^	2.5 (0.92 −4.49)	1.4 (0.47 −2.56)	n.s.
CD4^+^PD-1^+^	0.38 (0.00 −2.67)	0.31 (0.1 −2.10)	n.s.
CD8^+^PD-1^+^	0.06 (0.00 −0.09)	0.04 (0.02 −0.15)	n.s.
CD19^+^PD-L1^+^	0.05 (0.00 −0.07)	0.02 (0.00 −0.08)	n.s.
CD14^+^PD-L1^+^	1.20 (0.30 −3.34)	0.55 (0.28 −1.10)	n.s.

Percentages of Aβ_42_-stimulated cells are shown; median, interquantile ranges, and statistical significance are indicated.AD, Alzheimer’s disease; aMCI, amnestic mild cognitive impairment; IL, interleukin; BDNF, brain-derived neurotrophic factor; ROR, retinoid acid-related orphan receptor; T-bet, T-box expressed in T cells; IFN-**γ**, interferon-gamma; TGF- β, transforming growth factor-beta; PD-1, programed death-1; PD-L1, programed death-1 ligand.(*) *p*-Values refer to Mann–Whitney-U, significance level *p* < 0.05.In all cases, background results (i.e., % of cytokines secreting or PD-expressing cells in PBMC incubated with non-immunogenic peptides) were subtracted from the values shown.

#### Cytokine-producing CD14+ cells in Aβ_42_-peptide-stimulated cell cultures

The immune response is mediated by multiple cell types. To better approach the *in vivo* situation we examined IL-6-, IL-10-, IL-12p35-, IL-23-, and TGFβ-expressing CD14+ monocytes in peripheral mononuclear cell cultures that had been stimulated with the non-immunogenic or with the Aβ_42_-peptide. Results indicated that Aβ_42_-stimulated TGF-β-producing CD14+ cells were significantly decreased in AD compared to aMCI (*p* = 0.003) (Table 3).

#### PD-1-expressing CD4+ and CD8+ T lymphocytes and PD-L1-expressing CD14+ and CD19+ cells in Aβ_42_-peptide-stimulated PBMC

PD-1 on T lymphocytes binds PD-L1 on the surface of antigen presenting cells (APC) resulting in the dampening of adaptive immune-response via tolerization or apoptosis of antigen-specific T lymphocytes. Aβ_42_-stimulated lymphocytes and monocytes (APC) were analyzed in PBMC isolated from the peripheral blood of patients. Results showed that both PD-1- and PD-L1-expressing cells were reduced in AD compared to aMCI individuals, even if the differences did not reach statistical significance (Table 3). Again, no differences were observed when cells were incubated with the non-immunogenic peptides (data not shown).

### Correlations between MRI and immunologic parameters

Correlations were sought between the FA values that were significantly different between groups (CC2–CC3) and immunological parameters; separate analyses were performed for each group. Results showed significantly positive correlations in aMCI between the FA values for both CC2 and CC3 and circulating Aβ_42_-stimulated CD4+/BDNF+, CD8+/BDNF+, and CD14+/PD-L1+ cells (Table 4). These correlations were not maintained in AD patients in whom, instead, an important positive correlation was detected between the CC2 FA and Aβ_42_-stimulated CD4+/IL-21+ (inflammatory) lymphocytes (*R*_Sp_ = −0.41).

**Table 4:** **Correlations between MRI (DTI-metrics) and immunological parameters**.

Variable	With variable	*R*_Sp_ (95% C.L.)	*P* value
**aMCI**			
FA_CC2	CD14+/PD-L1+	0.53 (0.01; 0.82)	0.0389
FA_CC3	CD14+/PD-L1+	0.66 (0.21; 0.87)	0.0057
FA_CC2	CD4+/BDNF+	0.64 (0.08; 0.88)	0.0226
FA_CC3	CD4+/BDNF+	0.62 (0.05; 0.88)	0.0284
FA_CC2	CD8+/BDNF+	0.69 (0.16; 0.90)	0.0116
FA_CC3	CD8+/BDNF+	0.81 (0.40; 0.94)	0.0008
**AD**			
FA_CC3	CD4+/IL-21+	−0.41 (−0.68; −0.02)	0.0346

AD, Alzheimer’s disease; aMCI, amnestic mild cognitive impairment; CC, corpus callosum; FA, fractional anisotropy; *R*_Sp_, Spearman correlation coefficient and 95% confidence limits performed by Fisher’s *Z* transformation.

## Discussion

We investigated possible interactions among peripheral immunological parameters and brain disconnection in AD and aMCI individuals using DTI-based tractography. Analyses on brain disconnection focused on the CC, which appears as an ideal candidate for the investigation of WM damage in the different stages of the AD disease, due to its particular susceptibility to atrophy (Thomann et al., 2006; Chaim et al., 2007). This structure was evaluated in its seven different parts, allowing the definition of a valued topographic distinction of WM damage in different stages of AD pathology.

MRI results showed that different WM damage pattern are seen in aMCI and AD. Thus DTI analysis on FA indicated that the frontal CC subregions are preserved in the prodromal stage of the pathology (higher FA values on FA CC2–CC3 in aMCI), and MD values were still preserved in aMCI in almost the whole CC (higher MD values in AD only in CC7). This appears interesting when considering the different type of information provided by the two DTI indices: MD is more fit at analyzing extracellular volume alterations whereas FA better investigates impairments in the axonal integrity (Werring et al., 1999; Rovaris et al., 2005), independently of the gross tissue loss (Burzynska et al., 2010). When these indexes are analyzed together, lower FA and higher MD values suggests a loss of cortical regions related to the phenomenon of the anterograde Wallerian degeneration (Xie et al., 2006; Damoiseaux et al., 2009) whereas areas characterized by lower FA without particular changes on MD indicate the presence of a possible myelin breakdown process related to amyloid and tau deposits (Bartzokis, 2011; Lu et al., 2013). Results herein indicate that in aMCI brain disconnection mechanism are mainly due to myelin breakdown (lower FA without increase in MD). Interestingly, this process is located in almost the whole CC with the exclusion of frontal and parietal CC regions. This finding appears in line with previous results (Parente et al., 2008; Di Paola et al., 2010; Agosta et al., 2011; Preti et al., 2012), describing AD as part of a *continuum* of clinical and biological phenomena with a posterior to anterior pathophysiological evolution. This early damage of the posterior CC subregions in AD reflects the accumulation of proteinaceous deposits, such as Aβ and tau, involving initially the medial-temporal regions (Brun and Englund, 2002). These results are consistent with MRI-GM structural data and with the well-known pattern of distribution of neuropathological changes typical of AD neurodegeneration, which starts from medial-temporal regions then spreads to medial-parietal cortex and orbitofrontal regions and finally to other neocortical association areas (Busatto et al., 2008).

Results of immunological analyses performed in the same individuals showed that, upon Aβ_42_-stimulation, BDNF-producing CD4+ and TGF-β-producing CD14+ were significantly reduced, whereas IFNγ-producing and T-bet expressing CD8+ T cell were increased in AD compared to aMCI. These immune alterations were detected only *in vitro* Aβ_42_-stimulated cell cultures suggesting that they are specific for the aMCI-AD spectrum. TGF-β and BDNF are cytokines endowed with anti-inflammatory and neuroprotective function, even though recently these roles have become ambiguous. In particular TGF-β is a pleiotropic cytokine characterized by mutually exclusive regulatory and inflammatory activities that are cellular and environmental-context dependent. When the inflammatory properties prevail, TGF-β induces the differentiation of TH17 cells (Sanjabi et al., 2009). The observation that in aMCI TH17-produced cytokines (IL-17, IL-21, IL-22) were not detected seems to indicate that the biological significance of the increased production of TGF- β seen in these patients is anti-inflammatory. In AD the recruitment of peripheral monocytes/macrophages within the CNS could contrast the formation and/or reduce the extension of β-amyloid plaques via multiple mechanisms mediated by innate and acquired immunity (Tan et al., 1999a,b, 2002a; Town et al., 2001, 2005a,b, 2008; Townsend et al., 2005; Hawkes and McLaurin, 2009). Thus, it has previously been suggested that innate immune responses, possibly triggered by TLR activation, play a role in the AD-associated neuroinflammation (Fassbender et al., 2004; Fiala et al., 2007; Walter et al., 2007; Malm et al., 2010; Liu et al., 2012; Saresella et al., 2014). Notably, in AD patients T cell in peripheral blood and infiltrating the brain are indeed activated and display memory phenotype (Shalit et al., 1995; Lombardi et al., 1999, 2004; Tan et al., 2002b; Togo et al., 2002); levels of IL-6 in plasma (Singh and Guthikonda, 1997), augmented production of IFN-γ and TNF-α by natural killer (NK) cells (Solerte et al., 2000), and an increase in IL-1β associated with a concomitant decrease in IL-10 have been observed in AD (Arosio et al., 2004).

Activated T lymphocytes can nevertheless also mediate neuroprotection and promote neurogenesis by secreting BDNF. BDNF production was increased in aMCI; these results are is in line with data showing higher BDNF serum levels in preclinical stages of Alzheimer’s disease (Laske et al., 2006; Angelucci et al., 2010) and with post-mortem examinations of AD brains indicating a significant increase of BDNF concentration in hippocampus and parietal cortex (Durany et al., 2000) and of its receptor TrkB in astrocytes and senile plaques (Connor et al., 1997). BDNF was recently suggested to also mediate neurodegeneration secondarily to its ability to induce nitric oxide production by astrocytes (Colombo et al., 2012). Nevertheless, the observation that in aMCI a correlation was detected between BDNF and preserved brain morphology seems to confirm a protective role for this cytokine. Notably, the correlations between MRI parameters and BDNF secreting immune cells were lost in AD in whom, instead, brain atrophy was positively correlated with IL-21-producing CD4+ T cells. This seems biologically important considering that IL-21 is a potent proinflammatory cytokine that plays a role in autoimmunity, including multiple sclerosis, and whose biological properties include the proliferation of T cells proliferation, the stimulation of NK activity, and the functional dampening of T reg cells (Leonard and Spolski, 2005; Spolski and Leonard, 2008; Raveney et al., 2013). Confirming the presence of inflammation is AD are also the results indicating that TH1-driven IFNγ production by T-bet-expressing (TH1) CD8 + T lymphocytes was detected in these patients.

The preservation of brain morphology seen in aMCI was positively correlated with PD-L1 expressing CD14+ cells as well. PD-L1 binds PD-1 and this interaction dampens immune responses, harnessing inflammation. Recent results showed that the expression of these molecules is significantly reduced in AD and aMCI, thus, in these diseases, fewer PD-1-expressing CD4+ T cells bind reduced quantities of PD-L1 molecules on the surface of CD14+ APC. The interaction between PD-1 and PD-L1 regulates the reciprocal balance between T cell tolerance and activation, and recent data show that when PD-1 is engaged, the earliest events in T cell activation are blocked (Parry et al., 2005), indicating that PD-1 is a highly effective negative regulator of T-cell activation even when modestly expressed on T cell surface (Riley, 2009). Ligation of PD-L1 limits effectors’ cells responses and prevents the triggering of immune-mediated tissue damage (Selenko-Gebauer et al., 2003; Keir et al., 2007; Sharpe et al., 2007). This antigen-specific phenomenon is achieved via the generation of IL-10, a powerful inflammation-dampening cytokine, and/or by the limitation of the proliferation of antigen-specific cells secondarily to the apoptosis of such cells (Dong et al., 2002; Dong and Chen, 2003; Trabattoni et al., 2003). IL-10 was comparable in AD and aMCI, suggesting that modulation of apoptosis could be mechanism associated with the preservation of brain tissues seen in aMCI. The positive correlations seen in these individuals between CC2 and CC3 and PD-L1 expressing immune cells could speculatively be justified as follows: more Aβ specific cells would undergo apoptosis secondarily to more frequent PD-1/PD-L1 interactions; this would results in a reduced degree of neuroinflammation and, as a consequence, a better preservation of cortical architectures. This mechanism is lost in AD, leading to impairments of such structures.

These immunological results stem from analyses performed on circulating immune cells, in the attempt to define immunologic diagnostic and prognostic correlates of disease that would be easily monitored and reproduced. The fact that we used peripheral blood lymphocytes instead than cells circulating in the cerebro-spinal fluid (CSF) could apparently weaken our results. Because of ethical constrains, CSF examination could be performed in our patients only when necessary to exclude reversible causes of dementia. Nevertheless, the observations that immune cells continuously re-circulate throughout the body and migrate across the blood-brain barrier (BBB), and that such barrier is permeable to cytokines, lend support to our results.

In conclusion, the results herein seem to support the hypothesis that a disconnection syndrome in aMCI and AD could correlates with WM myelin breakdown caused by a neuroinflammation as pathophysiological substrate. The combination of DTI and neuroinflammation parameters might define possible surrogate biomarkers that could be useful in longitudinal studies and in clinical and pharmacological trials.

## Conflict of Interest Statement

The authors declare that the research was conducted in the absence of any commercial or financial relationships that could be construed as a potential conflict of interest.

## Footnotes

^1^http://dbm.neuro.uni-jena.de/vbm/

^2^http://www.fil.ion.ucl.ac.uk/spm/

^3^http://www.mathworks.it/products/matlab

^4^http://www.fmrib.ox.ac.uk/fsl/

^5^www.trackvis.org
